# Implication of *N*-Methyl-d-Aspartate Receptor in Homocysteine-Induced Age-Related Macular Degeneration

**DOI:** 10.3390/ijms22179356

**Published:** 2021-08-28

**Authors:** Yara A. Samra, Dina Kira, Pragya Rajpurohit, Riyaz Mohamed, Leah A. Owen, Akbar Shakoor, Ivana K. Kim, Margaret M. DeAngelis, Nader Sheibani, Mohamed Al-Shabrawey, Amany Tawfik

**Affiliations:** 1 Department of Oral Biology and Diagnostic Sciences, Dental College of Georgia, Augusta University, Augusta, GA 30912, USA; yaraadel@mans.edu.eg (Y.A.S.); dkira@augusta.edu (D.K.); prajpurohit@augusta.edu (P.R.); 2 James and Jean Culver Vision Discovery Institute, MCG, Augusta University, Augusta, GA 30912, USA; 3 Department of Biochemistry, Faculty of Pharmacy, Mansoura University, Mansoura 35516, Egypt; 4 Department of Physiology, Medical College of Georgia (MCG), Augusta University, Augusta, GA 30912, USA; rmohamed@augusta.edu; 5 Department of Ophthalmology, Jacobs School of Medicine and Biomedical Engineering, SUNY-University at Buffalo, Buffalo, NY 14214, USA; leah.owen@hsc.utah.edu (L.A.O.); mmdeange@buffalo.edu (M.M.D.); 6 Department of Population Health Sciences, University of Utah School of Medicine, Salt Lake City, UT 84108, USA; 7 Department of Ophthalmology and Visual Sciences, University of Utah School of Medicine, Salt Lake City, UT 84132, USA; akbar.shakoor@hsc.utah.edu; 8 Retina Service, Harvard Medical School, Massachusetts Eye and Ear, Boston, MA 02114, USA; Ivana_Kim@meei.harvard.edu; 9 VA Western New York Healthcare System, Buffalo, NY 14215, USA; 10 Department of Ophthalmology, Visual Sciences and Biomedical Engineering, University of Wisconsin School of Medicine and Public Health, Madison, WI 53726, USA; nsheibanikar@wisc.edu; 11 Department of Foundational Medical Studies and Eye Research Center, Oakland University William Beaumont School of Medicine, Rochester, MI 48309, USA; malshabrawey@oakland.edu; 12 Eye Research Institute, Oakland University, Rochester, MI 48309, USA

**Keywords:** *N*-methyl-d-aspartate receptor, homocysteine, age-related macular degeneration, blood retinal barrier, cystathionine-β-synthase, mouse

## Abstract

Age-related macular degeneration (AMD) is a leading cause of vision loss. Elevated homocysteine (Hcy) (Hyperhomocysteinemia) (HHcy) has been reported in AMD. We previously reported that HHcy induces AMD-like features. This study suggests that *N*-Methyl-d-aspartate receptor (NMDAR) activation in the retinal pigment epithelium (RPE) is a mechanism for HHcy-induced AMD. Serum Hcy and cystathionine-β-synthase (CBS) were assessed by ELISA. The involvement of NMDAR in Hcy-induced AMD features was evaluated (1) in vitro using ARPE-19 cells, primary RPE isolated from HHcy mice (CBS), and mouse choroidal endothelial cells (MCEC); (2) in vivo using wild-type mice and mice deficient in RPE NMDAR (*NMDAR^R^*^−/−^) with/without Hcy injection. Isolectin-B4, Ki67, HIF-1α, VEGF, NMDAR1, and albumin were assessed by immunofluorescence (IF), Western blot (WB), Optical coherence tomography (OCT), and fluorescein angiography (FA) to evaluate retinal structure, fluorescein leakage, and choroidal neovascularization (CNV). A neovascular AMD patient’s serum showed a significant increase in Hcy and a decrease in CBS. Hcy significantly increased HIF-1α, VEGF, and NMDAR in RPE cells, and Ki67 in MCEC. Hcy-injected WT mice showed disrupted retina and CNV. Knocking down RPE NMDAR improved retinal structure and CNV. Our findings underscore the role of RPE NMDAR in Hcy-induced AMD features; thus, NMDAR inhibition could serve as a promising therapeutic target for AMD.

## 1. Introduction

Age-related macular degeneration (AMD) is a leading cause of vision loss in people over 60 [1,2]. The number of AMD patients in 2020 was around 196 million and is expected to reach 288 million by 2040. Around 11 million new AMD patients are diagnosed every year in the United States [3]. The United States spends around USD 255 billion for AMD health care. AMD is the primary cause of visual disability in the developed world and third globally [4]. AMD causes negative effects on social and economic life such as lack of productivity, high treatment costs, and poor quality of life. Therefore, it is essential these days to find a new therapy for AMD. The major treatment options that have been developed in the last decades to treat the wet form of AMD are targeting the angiogenesis through anti-VEGF therapy. However, these AMD treatments are still unable to cure AMD; patients require unlimited treatment and do not regain their vision.

Recently, elevated homocysteine (Hcy) in relation to AMD has gained special attention in numerous clinical studies, suggesting a link between increased serum Hcy and the incidence of AMD [5,6,7,8]. Furthermore, our work reported the direct impact of excess Hcy (known as Hyperhomocysteinemia (HHcy)) on the retinal pigment epithelium (RPE) structure, barrier function, and induced choroidal neovascularization (CNV) in mice [9]. We also reported that HHcy increased VEGF levels in the retina [10] and the angiogenic potential of the retinal endothelial cells in vitro [11]. Understanding the molecular mechanism by which Hcy contributes to the pathogenesis of AMD remains a critical barrier in proposing Hcy as a biomarker and/or a therapeutic target for the treatment of AMD.

It has been demonstrated that *N*-methyl-d-aspartate (NMDA) receptor activation could be a possible mechanism of HHcy-induced ganglion cell death in the retina during diabetic retinopathy (DR) [12,13,14]. NMDAR has been identified as a receptor for Hcy in neurons [15]. NMDAR is located on the cerebral endothelium and implicated in increasing the permeability via glutamate-induced damage to endothelial cell (EC) integrity and in disrupting tight junction proteins [16,17]. Recently, we found that Hcy activated retinal endothelial NMDAR, resulting in BRB breakdown. Therefore, the inhibition of NMDAR could be a therapeutic target in retinal diseases related to HHcy such as AMD and DR [18]. The current study proposes the activation of NMDAR in RPE cells as an underlying target in HHcy-induced AMD pathology.

It is well-established that NMDAR plays a key role in brain trauma and neurodegenerative disorders [19,20]. Likewise, it has been found that Hcy treatment leads to an increase in NMDAR expression in brain microvascular ECs. Moreover, glutamate treatment increased NMDAR expression and RPE proliferation in cultured primary rat RPE cells, suggesting that NMDAR activation promotes the proliferation of RPE cells [21].

Retinal and choroidal neovascularization are the chief causes of major visual impairment. Therefore, understanding the different factors involved in neovascularization is essential in the development of novel treatments for visual impairment. The current study aims to assess the underlying molecular mechanisms of Hcy-induced RPE dysfunction. We propose that NMDAR activation in the RPE cells by HHcy plays a fundamental role in AMD induction, and that blocking NMDAR in RPE could be a novel therapeutic target for patients with AMD. We also generated mice deficient in NMDAR in the RPE cells (*NMDAR^R^*^−/−^) and examined whether knocking down the NMDAR in RPE cells has the ability to prevent Hcy-induced CNV and BRB permeability in AMD.

## 2. Results

### 2.1. Measurements of Homocysteine and CBS Enzyme Levels

**Figure 1:**
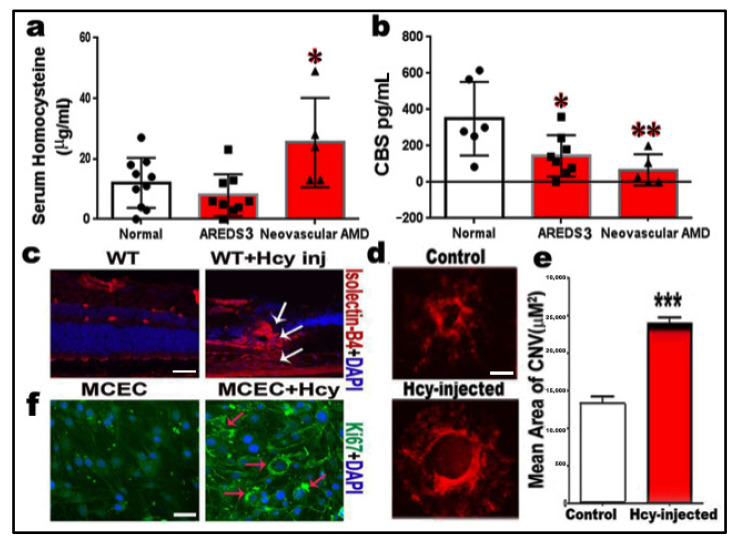
Homocysteine promotes angiogenesis and induces choroidal neovascularization (CNV). (**a**) ELISA measurement of serum Hcy showing a significant increase in patients with neovascular AMD. (**b**) ELISA measurement of serum CBS enzyme showing a significant decrease in patients with neovascular AMD. (**c**) Intravitreal injection of Hcy in wild-type mice induced choroidal neovascularization (CNV), as shown in retinal sections stained with isolectin-B4 (Arrows) (*n* = 6 mice per group). (**d**) Flat-mount retina stained with isolectin-B4. (**e**) Statistical analysis for the CNV size, showing that Hcy significantly increased the extent of laser-induced CNV in wild-type mice. (**f**) Immunofluorescence staining showing a marked increase in the immunoreactivity of the proliferation factor Ki67 in cultured mouse choroidal endothelial cells (green) by Hcy treatment (*n* = 4). Calibration bar: 50 µm; * *p* < 0.05, ** *p* < 0.01, and *** *p* < 0.001. Symbols (dark circles; represent number of normal patients, squares, represent number of AREDS patients and the triangles; represent the number of neovascular AMD patients).

Hcy and CBS enzyme levels were assessed in the serum of AMD patients and the normal donors as control. Hcy level was significantly (*p* < 0.05) increased in the serum of neovascular AMD donors as compared to normal control (*p* < 0.05) (Figure 1a). Furthermore, CBS enzyme level was significantly decreased in AREDS3 (representing intermediate AMD) and neovascular AMD patients as compared to normal control (*p* < 0.05) (Figure 1b). This data suggested elevated Hcy levels and impaired Hcy clearance in AMD patients, especially neovascular AMD patients.

### 2.2. Homocysteine Promotes Angiogenesis and Induction of Choroidal Neovascularization (CNV)

To study the effect of Hcy on CNV induction, we examined the CNV in the retinal frozen section and retinal flat-mounts of wild-type mice (C57-BL6) injected intravitreally with/without Hcy. Retinal section/flat-mounts were evaluated by immunofluorescence staining for vascular marker, isolectin-B4 (red). The results showed that Hcy-injected wild-type mice highly expressed isolectin-B4 and showed the development of blood vessels extending from the area of the choroid to the inner retina (white arrows, Figure 1c) as compared to the control wild-type mice, which revealed a normal retinal vascular pattern. Retinal flat-mounts of mice that were exposed to laser induction with/without the intravitreal injection of Hcy were stained with isolectin-B4 to examine the effect of Hcy on the size and extent of laser-induced CNV; this showed that Hcy significantly increased the extent of laser-induced CNV in wild-type mice (Figure 1d,e). Moreover, to confirm the effect of Hcy treatment on the proliferation of choroidal endothelial cells, we examined the expression of the Ki67 proliferation factor in cultured mouse choroidal endothelial cells (MCEC), and results showed a marked increase in Ki67 expression (green) in MCEC by Hcy treatment (Figure 1f).

### 2.3. Homocysteine Activates HIF-1α and VEGF in RPE Cells

**Figure 2:**
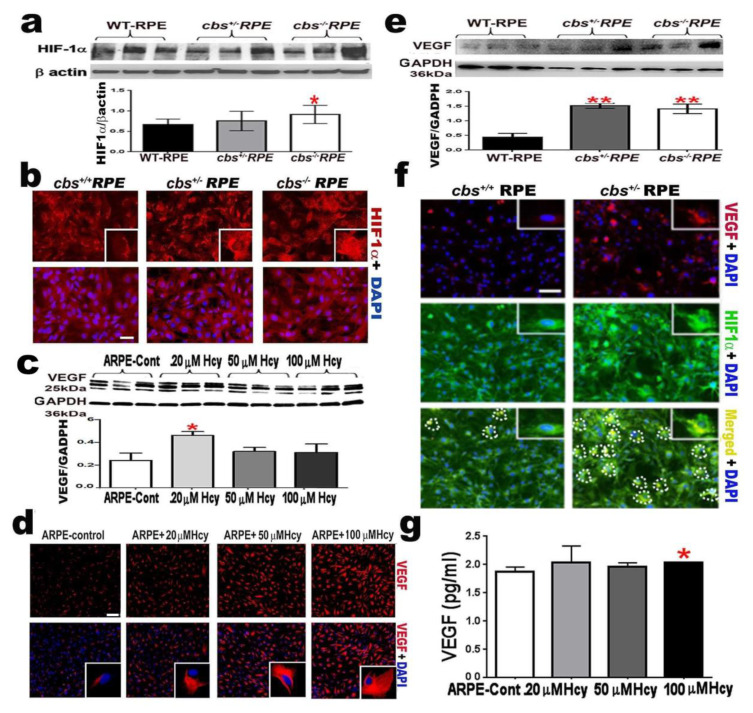
Homocysteine promotes angiogenic factors (**a**) Western blotting of HIF-1α expression in primary RPE cells isolated from mice model of elevated Hcy (wild-type *cbs*^+/+^, *cbs*^+/−^, and *cbs*^−/−^) showing a significant increase in HIF-1α expression in mice with marked HHcy; β-actin was used as loading control. (**b**) Immunofluorescence staining for HIF-1α (red) and nuclear staining (DAPI, blue) for primary RPE cells isolated from *cbs*^+/+^, *cbs*^+/−^, and *cbs*^−/−^ mice. Calibration bar: 50 µm. (**c**) Western blotting of VEGF expression in ARPE-19 treated with different concentrations of Hcy (20, 50, and 100 µM representing mild/moderate and sever elevation of Hcy); GADPH was used as a loading control. (**d**) Immunofluorescence staining for VEGF (red) and nuclear staining (DAPI, blue) for ARPE-19 treated with different concentrations of Hcy (20, 50, and 100 µM). Calibration bar: 100 µm. (**e**) Western blotting of VEGF expression in primary RPE cells isolated from wild-type *cbs*^+/+^, *cbs*^+/−^, and *cbs*^−/−^ mice. GADPH was used as a loading control. (**f**) Immunofluorescence staining for VEGF (red), HIF-1α (green), and nuclear staining (DAPI, blue) for RPE cells isolated from *cbs*^+/+^, *cbs*^+/−^ mice. Calibration bar: 50 µm. (**g**) ELISA evaluation of VEGF levels in ARPE-19 treated with different concentrations of Hcy (20, 50, and 100 µM). (*n* = 6 mice per group) for cells (*n* = 4). * *p* < 0.05, ** *p* < 0.01.

RPE cells are an important source of angiogenic factors in the retina. To further study the involvement of RPE in Hcy-induced angiogenesis and CNV, the expression of hypoxia-inducible factor (HIF-1α), which is a common transcription factor for several angiogenic proteins [22], and its downstream regulator of angiogenesis vascular—endothelial growth factor (VEGF)—were evaluated in primary RPE cells isolated from wild-type *cbs*^+/+^, *cbs*^+/−^, and *cbs*^−/−^ mice. The HIF-1α level was significantly upregulated by Hcy, as shown in primary RPE isolated from *cbs*^+/−^ and *cbs*^−/−^ mice. When it was evaluated by Western blot analysis (Figure 2a) and immunofluorescence (Figure 2b), HIF-1α increased in the *cbs*^+/−^ mice RPE (representing mild/moderate HHcy) and significantly increased in *cbs*^−/−^ mice RPE (representing a marked increase in HHcy), which was also very evident in immunofluorescence staining (red).

VEGF level was evaluated in ARPE-19 cells treated with different concentrations of Hcy (20, 50, and 100 µM) and primary RPE cells isolated from wild-type *cbs*^+/+^, *cbs*^+/−^, and *cbs*^−/−^ mice. The VEGF level was evaluated using Western blot analysis (Figure 2c,e), ELISA (Figure 2g), and immunofluorescence staining, as shown in red (Figure 2d). Hcy significantly increased the VEGF level in both ARPE-19 cells treated with Hcy and primary RPE cells isolated from mice with HHcy (*cbs*^+/−^ and *cbs*^−/−^). Furthermore, the activation and co-localization of both HIF-1α (green) and VEGF (red) in primary RPE cells isolated from the mice with HHcy was confirmed by immunofluorescence staining (Figure 2f).

### 2.4. Homocysteine Activates NMDA Receptors in RPE Cells

**Figure 3:**
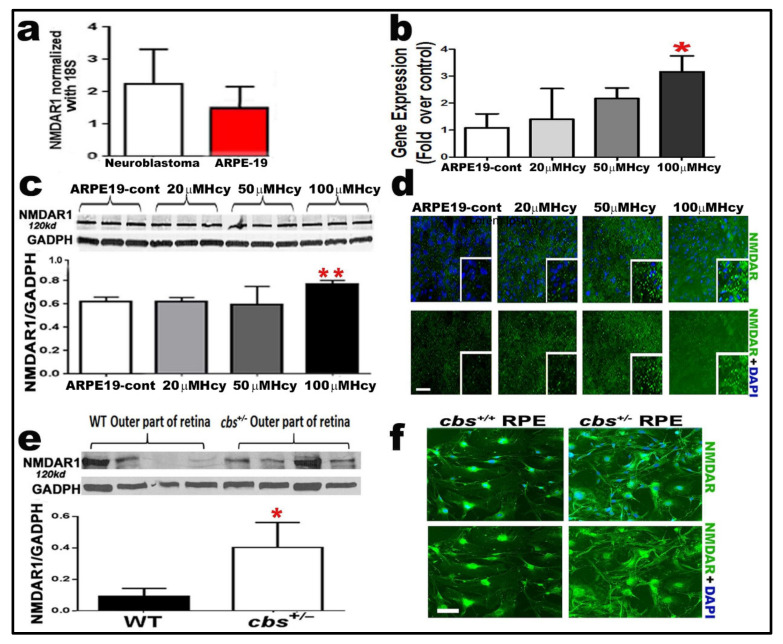
Evaluation of retinal epithelial cells (RPE) NMDAR expression in both in vivo and in vitro models of HHcy. (**a**) RT-qPCR analysis showing the expression of the NMDAR subunit NR1 in the human RPE (ARPE-19) cell line as compared to human neuroblastoma cells (ATCC CRL-2266) used as a positive control. (**b**) RT-qPCR analysis confirming NMDA receptor subunits NR1 (120 kD) in ARPE-19 cells and its activation by Hcy treatment (20 and 50 µM) as compared control untreated cells. (**c**) Western blot analysis showing the expression of the NMDAR subunit NR1 in human RPE (ARPE-19) treated with different concentrations of Hcy (20, 50, and 100 µM Hcy). GADPH was used as a loading control. (**d**) IF analysis showing the increased expression of NMDAR1 (green) in ARPE-19 treated with different concentrations of Hcy (20, 50, and 100 µM Hcy). (**e**) Western blot analysis showing the expression of NMDAR in the outer retina (containing mainly RPE cells) of the WT mice and *cbs*^+/−^ mice. GADPH was used as a loading control. (**f**) IF analysis showing the increased expression of NMDAR1 (green) in primary RPE cells isolated from *cbs*^+/−^ mice (*n* = 6 mice per group) for cells (*n* = 4). Calibration bar: 50 μm; * *p* < 0.05 and ** *p* < 0.01.

NMDAR1 expression was assessed in the human RPE (ARPE-19) cell line at both the gene and protein levels. The gene expression of NMDAR was assessed by RT-qPCR, and human neuroblastoma cells were used as a positive control. Our results showed that the NMDAR1 gene was expressed in the ARPE-19 cell line (Figure 3a). And its expression was increased by Hcy treatment in a dose dependent manner (Figure 3b). The expression of NMDAR was further confirmed on the protein level by assessing the expression of NMDAR1 by WB and IF analyses in ARPE-19 cells treated with/without Hcy (20, 50, and 100 µM). WB and IF analyses showed that NMDAR1 is highly expressed in the ARPE-19 cell line and the expression was significantly increased by a 100 µM Hcy treatment (Figure 3c,d) as compared to the ARPE-19 control. Finally, to further confirm our results, the protein expression of NMDAR1 was examined in the outer retina (has a good amount of RPE cells) of the mice model of HHcy (*cbs*^+/−^) and compared to the retina of normal control mice by WB analysis (Figure 3e), and in primary RPE cells isolated from wild-type mice and *cbs*^+/−^ mice by immunofluorescence. The *cbs*^+/−^ mice showed significant expression of NMDAR1 in the outer retina and increased activation (green) in *cbs*^+/−^ RPE cells as compared to primary RPE cells isolated from wild-type mice (Figure 3f).

### 2.5. Mouse with Deletion/Inhibition of NMDAR in Retinal Pigmented Epithelia (NMDAR^R −/−^)

**Figure 4:**
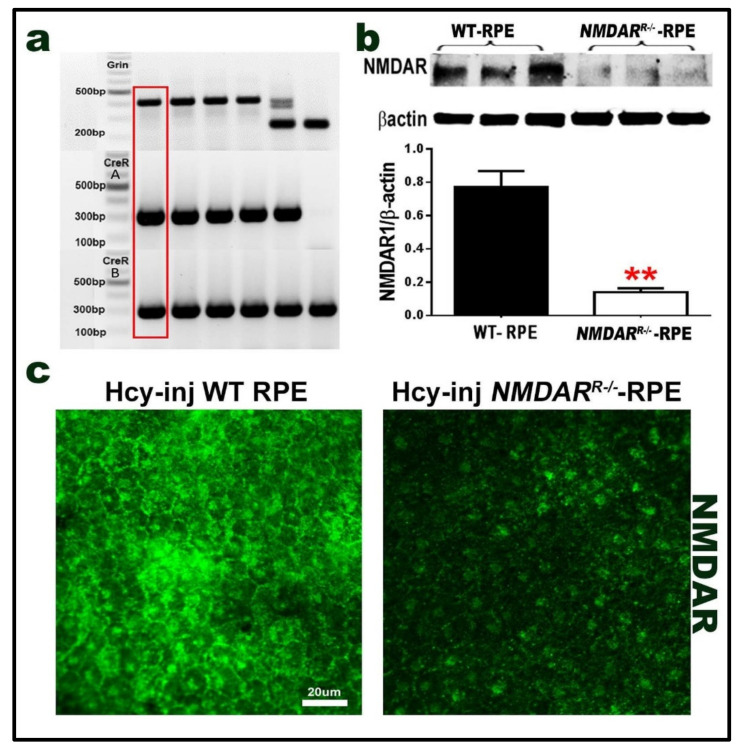
Mouse with the genetic deletion of NMDAR (*NMDAR^R^*^−/−^). A mouse deficient in NMDAR in the retinal epithelial cells (*NMDAR^R^*^−/−^) was generated in our lab by the backcrossing of B6.129S4-Grin1tm2Stl/J: (otherwise known as NR1flox, fNR1) with a CreR mouse. (**a**) PCR, genotyping analysis. Grin genotyping results: (*Grin*^+/+^) has only one band ~400 bp. (*Grin*^+/−^) has two bands ~400 bp and 232 bp. (*Grin*^−/−^) wild-type has a band at ~232 bp. CreR genotyping results: CreR reactionA^+^ has band at 300bp and CreR reaction B^+^ has band at 300 bp. The red-labeled (*NMDAr*^−/−*R*^) = *Grin*^+/+^ CreR reaction A^+^/CreR reaction B^+^. (**b**) Western blot analysis to confirm the reduced expression of NMDAR in primary RPE cells isolated from wild-type mice retina and primary RPE cells isolated from NMDAR^R−/−^, which showed a marked reduction in comparison to normal WT mice. β-actin was used as a loading control. (**c**) Immunofluorescence expression of NMDAR (green) of RPE flat-mounts from control wild-type and *NMDAR^R^*^−/−^ mice after the intravitreal injection of Hcy, which showed a marked reduction of NMDAR expression in the RPE layer of the mouse retina of the *NMDAR^R^*^−/−^mice as compared to control. (*n* = 6 mice per group) Calibration bar: 20 µm. ** *p* < 0.01.

We previously reported the involvement of endothelial NMDAR in HHcy-induced BRB dysfunction [18]. The current study aimed to further examine the involvement of the NMDAR of RPE cells in the HHcy-induced features of AMD. We generated mice deficient in the RPE cells NMDAR (*NMDR^R^*^−/−^); mouse genotyping is shown in Figure 4a. The deletion of NMDAR was confirmed using the western blot analysis of NMDAR1 in primary RPE cells isolated from *NMDR^R^*^−/−^ mice, which revealed a significant decrease of NMDAR1 in *NMDAR^R^*^−/−^ mice as compared to wild-type mice (Figure 4b). Additionally, NMDAR deletion was further confirmed in the retinal RPE flat-mount isolated from wild-type mice and *NMDAR^R^*^−/−^ mice 72 h after intravitreal injection of Hcy. RPE flat-mounts revealed the high expression of NMDAR in wild-type mice (confirming receptor activation by Hcy) as compared to the *NMDAR^R^*^−/−^ mice flat-mounts, which showed a marked decrease in NMDAR activation by Hcy and indicating receptor deletion/inhibition (Figure 4c).

### 2.6. Effect of NMDAR Deletion in RPE Cells on Hcy-Induced BRB Dysfunction

**Figure 5:**
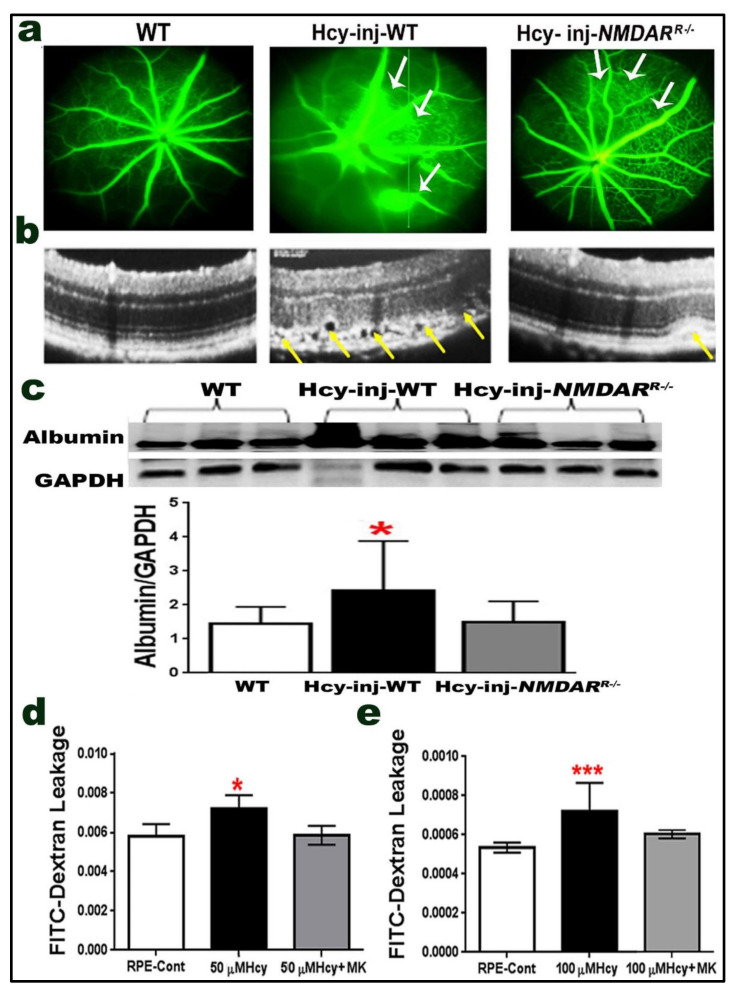
Knocking down the NMDAR in RPE cells (*NMDAR^R^*^−/−^ mice) protects retina from Homocysteine-induced blood retinal barrier (BRB) dysfunction and choroidal neovascularization (CNV). C57BL6 mice with/without Hcy-thiolactone intravitreal injection or genetic inhibition of NMDAR (*NMADR^R^*^−/−^) were evaluated 72 h after injection of Hcy by (**a**) FA evaluation, showing normal well-formed vessels in WT mice; however, angiograms for Hcy-injected mice retinas showed marked vascular leakage that appeared as diffused hyperfluorescence (white arrows), which was markedly reduced after knocking down NMDAR in RPE (*NMDAR^R^*^−/−^) mice. (**b**) OCT examination presenting normal appearance in the WT mice’s retina, marked interruption of retinal morphology with hypo-reflective subretinal lucency, focal hyper-reflective spots, and choroidal neovascularization (yellow arrows) in Hcy-injected mouse retinas. Knocking down NMDAR in RPE was able to reduce the retinal disruption via CNV and improved retinal structure after Hcy injection in *NMDAR^R^*^−/−^mice (yellow arrows), (*n* = 6 mice/group). (**c**) Reduced vascular leakage after knocking down NMDAR in RPE was confirmed by measuring the albumin leakage in the retinas by western blotting, which was significantly increased in the Hcy-injected mice eye but reduced to normal level in Hcy-injected *NMDAR^R^*^−/−^mice * *p* < 0.05. Improvement of retinal morphology and CNV induction was further evaluated by assessment of retinal thickness in Hcy-injected mice. (**d**) FITC dextran flux through the RPE monolayer, which revealed a significant increase in FITC dextran leakage in 50 µM Hcy-treated RPE cells and decreased by MK801 treatment. (**e**) FITC dextran flux through the RPE monolayer, which revealed a significant increase in FITC dextran leakage in 100 µM Hcy-treated RPE cells and decreased by MK801 treatment. (*n* = 6 mice per group) for cells (*n* = 4). * *p* < 0.05 and *** *p* < 0.001.

We previously reported that both the pharmacological (MK801) and the genetic inhibition of NMDAR in retinal endothelial cells (*NMDAR^E^*^−/−^ mouse) were able to reduce retinal damage and restore BRB induced by HHcy in vitro and in vivo [18]. After confirming the expression of NMDAR in retinal pigmented epithelial cells as well as its activation by Hcy, we wanted to examine whether blocking NMDAR in RPE cells (*NMDAR^R^*^−/−^ mouse) would rescue the retina from Hcy-induced blood retinal barrier (BRB) disruption and choroidal neovascularization (CNV) induction. We performed two functional studies, both in vivo and in vitro, using Fluorescein angiography (FA) and optical coherence tomography (OCT) in examinations for living mice and FITC dextran leakage assay in ARPE-19 cells treated with/without different concentrations of Hcy. FA and OCT were used to evaluate vascular leakage, retinal morphology, and CNV induction for living mice. Three groups of mice at age ~6–8 weeks were subjected to FA and OCT evaluation (wild-type (C57-BL6)) compared to wild-type and *NMDAR^R^*^−/−^ mice, 72 h after intravitreal injection of Hcy (200 µM). FA examination showed increased fluorescein leakage (white arrows) and disrupted retinal morphology in Hcy-injected wild-type mice as compared to wild-type control mice, suggesting decreased retinal vessel integrity and the impairment of BRB by Hcy. However, the genetic inhibition of NMDAR by knocking it down in RPE cells (*NMDAR^R^*^−/−^) was able to decrease fluorescein leakage (white arrows) and restore BRB (Figure 5a). OCT evaluation also showed a normal appearance in wild-type mice but a marked disruption on the RPE layer and CNV induction (yellow arrows) in the retinas of Hcy-injected wild-type mice. Moreover, knocking down NMDAR in RPE cells (*NMDAR^R^*^−/−^) improved retinal structure and CNV induction (yellow arrows) after Hcy injection (Figure 5b). Vascular leakage was confirmed by measuring the albumin leakage in the retinas via western blot analysis after perfusion with PBS solution and as previously described in our publications [9,18,23,24]. Quantification of data from western blotting showed significant increase in albumin leakage in the retina of Hcy-injected mice as compared to non-injected mice, and the albumin leakage was significantly decreased in *NMDAR^R^*^−/−^ mice injected with Hcy, suggesting that the blocking of NMDAR could restore the BRB and prevent retinal leakage induced by Hcy (Figure 5c).

To further study the effect of inhibition of NMDAR on the permeability of RPE cells treated with/without Hcy, an in vitro functional assay was performed through the pharmacological inhibition of NMDAR by MK801. We investigated whether Hcy induced permeability changes in FITC dextran flux through the ARPE-19 confluent monolayer. Hcy (50 and 100 µM) in the presence/absence of MK801 (25 µM) was added. Our data showed that Hcy treatment significantly increased FITC dextran leakage in the RPE cells monolayer, and that MK801 treatment significantly decreased the leakage and was able to restore the RPE barrier (Figure 5d,e).

### 2.7. Effect of NMDAR Deletion in RPE Cells on Hcy-Induced CNV and Retinal Thickness

**Figure 6:**
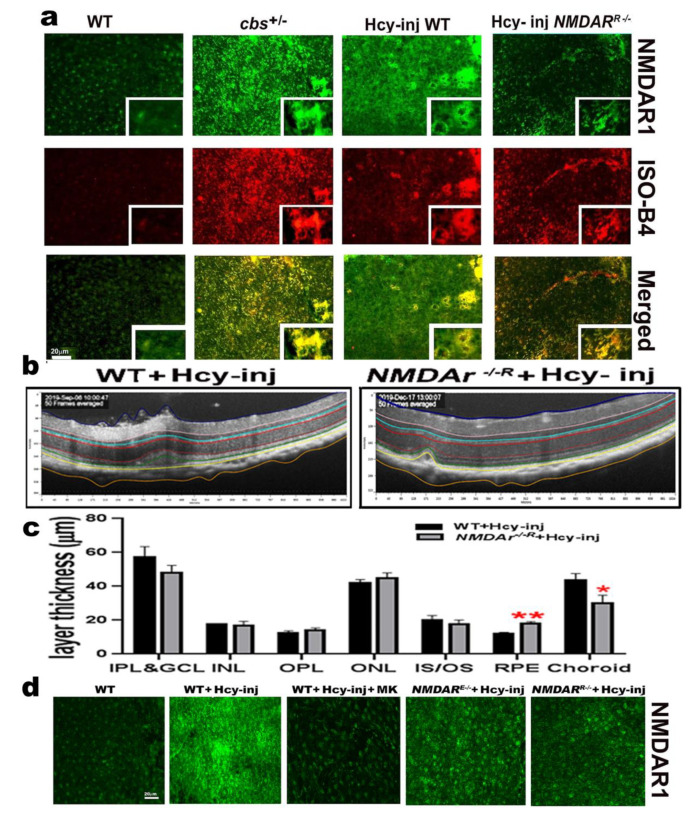
Deletion of NMDAR in RPE cells decreased Hcy-induced CNV in retinal flat mounts. (**a**) Immunofluorescence staining for RPE flat-mounts isolated from mouse retinas after one week of intravitreal injection of Hcy in WT and *NMDAR^R^*^−/−^ as compared to WT control non-injected mice and mice with HHcy (*cbs*^+/−^*)* stained with a vascular marker using Isolectin-B4 (red) and NMDAR1 (green). Hcy injection induced choroidal neovascularization and activation of NMDAR, which was more evident in both mice models of HHcy (*cbs*^+/−^ mice and Hcy-injected WT mice), while knocking down NMDAR in (*NMDAR^R^*^−/−^) was able to reduce CNV induction and NMDAR activation by Hcy injection. (**b**) OCT images and Insight^®^ software were used for assessment of the thickness of different retinal layers in wild-type and *NMDAR^R^*^−/−^mice 72 h after intravitreal injection of Hcy. (**c**) Analysis of retinal thickness of WT mice and *NMDAR^R^*^−/−^ mice injected with Hcy showed an improved RPE layer and decreased CNV size in *NMDAR^R^*^−/−^mice. (**d**) Outer retina flat-mounts stained with an antibody for NMDAR (green), confirming that both the pharmacological (MK801) and the genetic inhibition of NMDAR were able to block the Hcy activation of NMDAR. (*n* = 6 mice per group). Calibration bar: 20 µm; * *p* < 0.05, ** *p* < 0.01.

RPE flat-mounts isolated from mouse retinas after one week of intravitreal injection of Hcy were prepared according to our previously published method [9,18,23,24] and stained with immunofluorescence stain for the vascular marker using Isolectin-B4 (red) and NMDAR (green). Mounts showed that Hcy injection induced choroidal neovascularization and the activation of NMDAR, which was more evident in the HHcy mice (*cbs*^+/−^ mice and Hcy-injected mice) as compared to wild-type control non-injected mice and *NMDAR^R^*^−/−^ mice. Deletion of NMDAR in RPE cells was able to reduce the CNV induction and NMDAR activation by Hcy injection (Figure 6a). OCT images and Insight^®^ software were used for the assessment of the thickness of different retinal layers in wild-type and *NMDAR^R^*^−/−^ mice 72 h after intravitreal injection of Hcy. The average thickness of each layer in a 300mm section of wild-type mice and *NMDAR^R^*^−/−^ mice retina were compared. Analysis of retinal thickness showed that the RPE layer was significantly restored, and the choroid layer (via CNV induction) was significantly decreased in Hcy-injected *NMDAR^R^*^−/−^ mice as compared to Hcy-injected wild-type mice (Figure 6b,c). Outer retina flat-mounts were stained with an antibody for NMDAR (green), which confirmed that both the pharmacological (MK801) and the genetic inhibition of NMDAR were able to block the Hcy activation of NMDAR (Figure 6d).

### 2.8. Effect of Pharmacological and Genetic Intervention of NMDAR on Retinal Morphology and Vasculature after Laser Induction of Choroidal Neovascularization (CNV)

**Figure 7:**
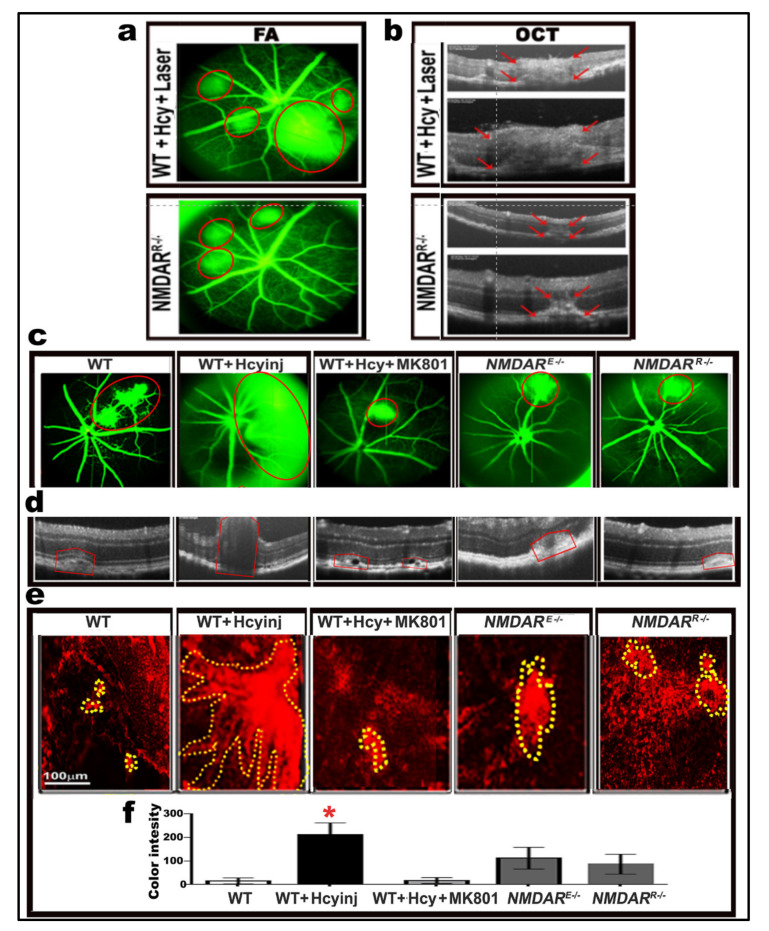
Knocking down the NMDAR protects retina after laser induction. (**a**,**b**) FA and OCT evaluation images of WT and NMDAR^R−/−^ mice injected intravitreally with Hcy, showing marked retinal disruption and CNV induction (red circles in FA and red arrow on OCT), which extended through the whole retina after laser induction in the WT mice injected with Hcy, while knocking down the NMDAR in RPE cells was able to reduce the CNV and protect the retina from the Hcy effect in Hcy-injected NMDAR^R**−/**−^ mice. (**c**,**d**) FA and OCT evaluation of retinal CNV after laser induction, showing that both the pharmacological (MK801) and the genetic inhibition of NMDAR (endothelial NMDAR^E−/−^ and RPE, NMDAR^R−/−^) were able to protect the retina from Hcy-induced CNV after exposure to laser burns (outlined by red in both FA and OCT). (**e**,**f**) RPE flat-mounts were prepared from the same mice groups for further assessments; mounts were stained for vascular marker isolectin-B4 (IB4) and imaged by All-in-One Fluorescence Microscope (BZ-X800), Keyence corporation. CNV areas are circled in yellow and CNV sizes were assessed by image J program quantitative images representing CNV sizes that confirmed the FA and OCT data. (*n* = 6 mice per group). Calibration bar: 100 µm. * *p* < 0.05.

First, we wanted to test the effect of NMDAR inhibition at the RPE level after the laser induction of CNV in mice (WT and NMDAR^R−/−^ injected intravitreally with 200 µM Hcy for 72 h). FA and OCT were measured after laser induction to evaluate the retinas of living mice and test whether knocking down the NMDAR in RPE cells (NMDAR^R−/−^ mice) protected the retina from laser-induced CNV. Hcy-injected wild-type mice showed marked retinal disruption and CNV induction, indicated as marked fluorescein leakage (red circles in FA) and red arrows in OCT, which extended through the whole retina after laser induction. However, knocking down the NMDAR in RPE cells was able to reduce the CNV and protect the retina in Hcy-injected NMDAR^R−/−^ mice (Figure 7a,b).

We previously reported that both the pharmacological (MK801) and the genetic inhibition of NMDAR in retinal endothelial cells (NMDAR^E−/−^ mouse) were able to reduce retinal damage and restore BRB induced by HHcy both in vitro and in vivo. We wanted to test and compare whether blocking NMDAR through the pharmacological (MK801) or genetic inhibition of NMDAR in the endothelial cells (NMDAR^E−/−^) or in the RPE cells (NMDAR^R−/−^) could rescue the HHcy-induced disruption of BRB and CNV induction. We performed a functional study in which five groups of mice were used at the age of ~6–8 weeks (WT, WT + intravitreal injection of Hcy, WT + intravitreal injection of Hcy + MK801 (IP, 25 µM) prior to Hcy injection), NMDAR^E−/−^ + intravitreal injection of Hcy and NMDAR^R−/−^ + intravitreal injection of Hcy). Hcy was injected intravitreally (200 µM) 48 h before laser induction. The mice were subjected to laser burns to induce CNV, followed by OCT and FA examination; FA and OCT confirmed our previously reported finding that Hcy induced retinal damage, BRB disruption, and CNV induction, which was more severe after the laser induction in the WT mice. However, both the pharmacological and the genetic inhibition of NMDAR were able improve retinal morphology and CNV induction, as indicated by the red circle on the FA images and the red arrows on the OCT images (Figure 7c,d).

RPE flat-mounts were collected from mice retina and immunostained for the vascular marker isolectin B-4 (IB4) to allow for the examination of the CNV extension and a quantitative measurement of the CNV. The microscopic examination and the colorimetric measurements confirmed the FA and OCT finding that both the pharmacological and genetic inhibition of NMDAR (either endothelial or RPE) were able to protect the retina from the harmful effects of Hcy on retinal morphology, BRB, and reduced Hcy-induced CNV (Figure 7e,f).

## 3. Discussion

The current study presents evidence suggesting that the activation of NMDAR as a possible mechanism of HHcy-induced BRB dysfunction, features of AMD and CNV induction. Our previous findings showed that HHcy produced AMD-like features in the RPE and induced CNV in vivo when it was injected into mice eyes, and in vitro in RPE cells treated with different concentrations of Hcy [9]. Data presented in the current study (1) showed the activation of NMDAR in RPE cells by HHcy in vivo and in vitro, (2) confirmed the activation of NMDAR by Hcy at the level of RPE cells and that the blocking of NMDAR attenuates Hcy-induced features of AMD, retinal hyperpermeability, and development of CNV, and (3) tested the blocking of NMDAR by pharmacological inhibition using MK801 and by molecular inhibition using RPE in NMDAR conditional knockout mice that were created in our lab (NMDAR^R−/−^).

The current study emphasizes the involvement of NMDAR in Hcy-induced outer BRB dysfunction, and Hcy-induced features of AMD and CNV induction. AMD is the most common cause of blindness in elderly people [25]. Generally, AMD is divided into two stages—early and late age-related maculopathy (ARM)—according to the International Classification and Grading System [26]. Advanced stage (late) AMD is further classified into two subtypes of AMD: non-neovascular (dry, atrophic) and neovascular (wet, exudative) types. Non-exudative AMD is characterized by the gradual loss of the RPE layer and the thinning of the retina, and only a few preventative/therapeutic measures are currently available for the dry type [27]. Conversely, exudative AMD is characterized by CNV and a subretinal neovascular fibrous tissue. CNV is responsible for 80% of AMD severe vision loss cases [28]. Anti-VEGF therapy has demonstrated great benefits for the wet type; however, issues such as non-responses or the need for repeated injections continue to occur [29]. The mechanisms of AMD progression are not completely understood. Accumulating evidence from many published clinical studies are consistent with our results, which revealed an association between elevated plasma Hcy level and the risk of AMD [6,30,31], predominantly the neurovascular (wet) AMD [27,32]. Furthermore, reported data from a case control study that was conducted in a tertiary eye care hospital with 32 diagnosed AMD patients supports our results, which showed that HHcy was significantly associated with the wet AMD patients but not with the dry AMD patients [33].

Previously, we reported the direct angiogenic effect of Hcy on human retinal endothelial cells (HREC) [11] and the activation of NMDA receptors in retinal endothelial cells by Hcy [18]. NMDAR activation was also reported to induce hyperpermeability by HHcy in retinal neurons and cerebral endothelial cells in several studies [16,34,35,36,37]. The study also demonstrated that Hcy level was significantly increased in the serum of neovascular AMD patients. This was associated with decreased Hcy clearance, as indicated by the decrease in the CBS enzyme level. Therefore, we have recognized HHcy as an important player in the pathogenesis of AMD. To explore the fact that HHcy is more involved in neovascular AMD, we tested the ability of Hcy to induce CNV after laser induction in wild-type mice with/without the intravitreal injection of Hcy. Our results showed that laser induction induced CNV in mice retina, which was significantly more severe in Hcy-injected mice as compared to non-injected mice.

An intact BRB is essential for the integrity of retinal structure and function. The breakdown of BRB has very bad effects on the vision. The BRB controls the nutrients and fluid movement between retinal tissues and ocular vascular beds. BRB is divided into inner BRB near the vitreous body and outer BRB near the choroids. Inner and outer BRB are responsible for preserving normal retinal microvascular homeostasis. The outer BRB is formed by tight junctions between RPE cells set on the Bruch’s membrane, separating RPE from the underlying choriocapillaris. On the other hand, the inner BRB is formed by tight junctions between RECs, which rest on a basal lamina separating them from pericytes and covered by foot processes of astrocytes and Müller cells [34].

The current study confirmed our previous finding both in vitro and in vivo. Our data showed that Hcy increased FITC-dextran leakage in RPE, fluorescein and albumin leakage as well as the development of CNV in mice eyes when Hcy was injected intravitreally. Furthermore, our results showed that blocking NMDAR at the RPE level either by pharmacological or molecular inhibition was able to buffer the effect of HHcy on the barrier function both in vitro and in vivo.

Hypoxia plays a key role in retinal ischemia and neovascularization, and it is the main stimulus for angiogenesis. Previously, we reported tissue hypoxia in cbs mice retinas (mice with HHcy) with the upregulation of VEGF and the development of neovascularization [10]. Cellular responses to hypoxia are mediated by hypoxia-inducible transcription factors (HIFs) that are stabilized in hypoxia and prompt the upregulation of many genes involved in angiogenesis, including VEGF [38,39,40]. HIF-1 is a nuclear protein which activates gene transcription in case of reduced oxygen tension. HIF-1 is a heterodimer composed of the HIF-lα and HIF-1β subunits. HIF-1β is constitutively expressed, while HIF-lα is induced and accumulates by hypoxia. HIF-1α, the HIF-1 heterodimer, increases the expression of angiogenesis and glycolysis genes. VEGF is the main angiogenic factor that is activated by HIF-1 to improve the hypoxic condition via the stimulation of vascular growth. This is consistent with the presented findings that HHcy activated HIF-1α and VEGF expression in the RPE cells and Ki67 in MCEC, indicating its proliferation. The increased production of the proangiogenic factor VEGF from RPE cells plays a major role in CNV formation [41,42]. Subsequently, the proangiogenic factor VEGF leads to the degradation of the extracellular matrix via the release of inflammatory mediators from endothelial cells. VEGF is known to cause a breakdown of the BRB and increase retinal vascular permeability and leakage [43,44].

Moreover, to test the angiogenic effect of Hcy, we tested the effect of Hcy on RPE and MCEC cells. Our results showed that Hcy treatment significantly increased the protein levels of both HIF-lα and VEGF in the ARPE-19 cell line and in primary RPE isolated from *cbs*^+/−^ mice as compared to control non-treated ARPE-19 cells and wild-type mice RPE. MCEC treated with Hcy showed the activation of its proliferation, indicated by the upregulation of Ki67. These results prove that angiogenesis is a part of the Hcy mechanism of BRB dysfunction and CNV induction.

We proposed that Hcy participates in the pathogenesis of AMD, and induces CNV and angiogenesis via the activation of the NMDA receptor. NMDA receptors are glutamate receptors that consist mainly of the GluN1 and GluN2 subunits. NMDA receptor activation requires the binding of neurotransmitter agonists to a ligand-binding domain (LBD) and the structural rearrangement of an amino-terminal domain (ATD). There is a similarity between the structure of Hcy and L-glutamate (the usual activator for the receptors) [34]. In congruence, Hcy was reported to bind to NMDAR, which is a glutamate receptor in neurons [15], and to activate NMDAR in cerebral endothelium, resulting in the disruption of tight junctions and blood–brain barrier (BBB) dysfunction. Therefore, NMDAR could be a target for therapeutic intervention in HHcy [16,17]. Additionally, several studies have suggested that the activation of NMDA receptors could be a possible mechanism of HHcy-induced retinal ganglion cell death [12,15,45]. Recently, it has been reported that hHcy-induced retinal endothelial cell dysfunction is mediated via the activation of the NMDAR signaling pathway [18].

The dramatic decline in cardiovascular mortality in the United States since 1950 may be attributable, in part, to the intended fortification of the food supply with vitamin B-6 and folic acid, apparently because of increased blood folate and decreased blood homocysteine, which is a well-known risk factor for cardiovascular diseases [46]. Furthermore, combined vitamin-B6, B12, and folate were reported to exert a neuroprotective effect against hypoxia in mice brain [47]. Our future plan is to test the effect of vitamins B12 and folic acid supplementation on Hcy-induced BRB dysfunction and CNV induction. However, the current study aimed to test the role of NMDAR in RPE cells in the pathogenesis of HHcy-induced AMD-like features. First, we tested the expression of the NMDA receptor in RPE cells. We assessed the NMDA receptor expression in both primary RPE isolated from *cbs* mice (*cbs*^+/+^ and *cbs*^+/−^, mice with HHcy) and the human retinal RPE cell line (ARPE-19). Our data showed that NMDAR is expressed in both the cbs-RPE and the human ARPE-19 cell lines. Furthermore, cbs^+/−^primary RPE cells showed higher expression of NMDAR than the wild-type (*cbs*^+/+^) RPE, indicating the activation of NMDAR by Hcy. Moreover, Hcy treatment (100 µM) significantly increased NMDAR1 in ARPE-19 cells as compared to untreated cells. These results confirmed the possibility that Hcy participates in the pathogenesis of AMD via the activation of the NMDA receptor in RPE cells. Consistent with our hypothesis, the involvement of NMDAR activation in RPE has been reported in many publications [21,48,49] and the NMDAR blocker Memantine was reported to have a protective effect on the ARPE-19 cells [48].

In order to prove our hypothesis in vivo, we generated mice deficient in NMDAR in the RPE cells (*NMDAR^R^*^−/−^) and performed more experiments to test if the deletion of NMDAR in RPE cells (1) would protect the retina from the harmful effects of Hcy, (2) would preserve the BRB indicated by retinal vascular leakage, (3) would protect from Hcy-induced CNV. For this purpose, living mice injected intravitreally with/without Hcy were exposed to FA and OCT examinations and albumin leakage assessment. FA and OCT results showed that knocking down the NMDAR in RPE cells decreased fluorescein leakage and CNV induction. Preservation of the BRB function was confirmed by the measurement of albumin leakage after the Hcy intravitreal injection of wild-type mice and *NMDAR^R^*^−/−^ mice retinas. Blocking NMDAR at the RPE level significantly decreased albumin leakage as compared to Hcy-injected wild-type mice retinas. Furthermore, when the mice were exposed to the laser induction of CNV, knocking down the NMDAR in RPE cells protected the retina and showed less CNV induction than Hcy-injected WT mice. This protective effect of knocking down the NMDAR in RPE cells was confirmed by measuring retinal thickness. The results showed that the RPE layer was significantly restored, while the choroid layer (CNV induction) was significantly decreased in Hcy-injected *NMDAR^R^*^−/−^ mice as compared to Hcy-injected wild-type mice. Therefore, these findings highlighted implication of the NMDA receptor in Hcy-induced AMD-like features.

In conclusion, the presented findings of the current study highlight new molecular mechanisms of HHcy-induced RPE dysfunction. Indeed, our data from in vivo and in vitro experiments demonstrate NMDAR activation as an underlying molecular target for Hcy in AMD retina. Unraveling these molecular targets help to achieve a better understanding and shed the light on novel therapeutic approaches for HHcy-induced retinal damage and CNV induction in AMD patients.

## 4. Materials and Methods

### 4.1. Animals

#### 4.1.1. Mouse with Deletion or Inhibition of NMDAR

We generated mice deficient in NMDAR in the RPE cells (NMDAR^−/−R^) by backcrossing floxed NR1 mice with C57BL/6-Tg (BEST1-cre) 1 Jdun/J mice (Jackson Lab, Bar Harbor, ME, USA) that express Cre recombinase under the control of the human bestrophin 1 (BEST) promoter in order to investigate the involvement of the NMDAR of RPE cells in the HHcy-induced features of AMD (Figure 4). These floxed NR1 mice allow for the deletion of the GluN1 subunit of the *N*-methyl-d-aspartate receptor in the Cre recombinase-expressing cell. This approach is useful in studying NMDAR and its downstream signaling molecules/pathways. Mice that are homozygous for this allele are viable, fertile, and normal in size, and do not display any gross physical or behavioral abnormalities. These mice were previously used to study the role of the NMDAR in remote memories and behavior [50,51], and our lab also generated NMDAR^E−/−^, endothelial cell conditional knockout mice used in studying the NMDAR1, its downstream signaling molecules/pathways, and its involvement in HHcy-induced BRB dysfunction [18].

#### 4.1.2. Mouse with HHcy

Pairs of cbs^+/−^ mice (B6.129P2-Cbstm1Unc/J; Jackson Laboratories, Bar Harbor, ME, USA) were bred to establish colonies of cbs^+/+^, cbs^+/−^, and cbs^−/−^ mice. Genotyping was performed according to the Jackson animal laboratory’s protocol. This was based on whether the mouse was heterozygous (cbs^+/−^) with one cbs copy, or homozygous (cbs^−/−^), which has no copies of the cbs enzyme. Therefore, the cbs^+/−^ mice had about a 4- to 7-fold increase in plasma Hcy level, ranging from mild to moderate retinal phenotype, with normal life span, and represented mild/moderate HHcy; the cbs^−/−^ mice had about a 30-fold increase in plasma Hcy, showed severe retinal phenotype, a short life span of ~3–5 weeks, and represented severe HHcy. Cbs mice have been used as a model of HHcy in our and others’ publications [9,11,18,23,24,52,53,54].

For the intravitreal injection of Hcy in mice, the procedure was the same as described in our previous publications [9,18,23,24]. A total of 1 µL was used as intravitreal injection volume to avoid an uncontrolled intraocular pressure increase. A 10X stock solution of l-homocysteine thiolactone hydrochloride (Sigma-Aldrich, St. Louis, MO, USA) was prepared in distilled water, and a working solution was prepared by diluting 1 µL of the stock solution (200 mM) in 100 µL of the phosphate buffer saline (PBS). To obtain 200 µM of the vitreal concentration of Hcy-thiolactone, 1 μL of this working solution was injected. PBS-injected control eyes showed normal retinal morphology with no apparent apoptosis within 7 days, demonstrating that the volume of the injected solution apparently did not result in significant pressure-induced damage in the retina.

All animals used in the current study were maintained in clear plastic cages, allowed to eat and drink ad libitum, and were subjected to standard laboratory conditions (12 h light/12 h dark cycles, temperature at 22–24 °C). All experimental procedures were performed according to the Public Health Service Guide for the Care and Use of Laboratory Animals (Department of Health, Education, and Welfare publication, NIH 80-23) and Augusta University guidelines (protocol number: 2014-0683, 11/20/2020), and followed the ARVO Statement for Use of Animals in Ophthalmic and Vision Research.

### 4.2. Measurement of Homocysteine and Cystathionine Beta-Synthase (CBS) Enzyme Levels

The concentration of Hcy and CBS enzyme levels were assessed in the serum of neovascular and non-neovascular AMD patients as compared to normal control using the Hcy Enzyme-Linked Immunosorbent Assay (ELISA) kit from Cell Bio Labs Inc (STA-670, San Diego, CA, USA) and the CBS ELISA assay kit from My BioSource (MBS700623, San Diego, CA, USA). The patients’ blood samples were provided by Dr. Margaret M. DeAngelis and were approved by the Institutional Review Board (IRB) at the University of Utah. The blood samples collected were allowed to clot in serum separator tubes (SST) for a minimum of 2 h at room temperature, preceding centrifugation at 1000× *g* for 15 min. The serum was collected and immediately assessed according to the protocol delivered with the kit. The readings were taken at 450 nm using an ELISA plate reader. Serum samples were taken from AMD patients (3 females and 2 males; age 83 ± 6.5) and controls (4 females and 6 males; age 74 ± 9.6); all donors’ ancestry was Caucasian European. Donors’ comorbidities (disease and normal) included dyslipidemia and hypertension, and the cause of death was myocardial infarction. Donor samples were collected, determined, managed, and phenotyped, as previously defined for the Utah protocol [55]. The clinically derived modified Age-Related Eye Disease Study severity grading scale (AREDS 1, AREDS 2, AREDS3 (intermediate), AREDS 4a (geographic atrophy), AREDS 4b (nAMD)) was used [56]. This protocol was approved by the IRB (IRB 00052879) at the University of Utah and conforms to the tenets of the Declaration of Helsinki.

### 4.3. Cell Culture

The human retinal pigmented epithelial cell line (ARPE-19) was obtained from American Type Culture Collection (ATCC, Manassas, VA, USA). ARPE-19 at passage 6–15 was cultured in a DMEM/F-12 growth medium (Thermo-Scientific, Wyman, MA, USA), supplemented with penicillin/streptomycin 1% and fetal bovine serum (FBS) 10%. At 80–90% confluency, the cells were serum starved overnight and then treated with Hcy (20 or 50 or 100 µM) or vehicle for 24 h. Then, the supernatant and/or the cells were harvested for further analyses.

### 4.4. Isolation and Culture of Primary Retinal Pigment Epithelium (RPE)

Wild-type, *cbs^+^*^/*−*^, *cbs^−^*^/*−*^*,* and NMDAR^R*−*/*−*^ mice (~3 weeks old) were used for the isolation of RPE cells as previously published [53,57]. Briefly, mouse eyes were enucleated and rinsed in a 5% povidone-iodine solution, then rinsed with sterile Hank’s Balanced Salt Solution (HBSS). Then, connective tissues were cleared away and eyes were placed in cold RPE cell culture medium (DMEM: F12), which was supplemented with 25% fetal bovine serum, 0.1 mg/mL gentamicin, 100 U/mL penicillin, and 100 µg/mL streptomycin. After that, the eyes were transferred to HBSS containing 19.5 U/mL collagenase and 38 U/mL testicular hyaluronidase and incubated at 37 °C for 40 min; this was followed by incubation in HBSS containing 0.1% trypsin (pH 8) at 37 °C for 50 min. The eyes were subsequently placed in a new dish with the RPE cell culture medium at 4 °C for at least half an hour. After that, the eyes were dissected, and isolated RPE cells were centrifuged at 1200× *g* for 10 min (Thermo Medilite Centrifuge, Thermo Scientific, Waltham, MA, USA), followed by the suspension and culture of RPE cells in an RPE cell culture medium (DMEM: F12) at 37 °C.

### 4.5. Isolation and Culture of Mouse Choroidal Endothelial Cells (MCEC)

MCEC were isolated and maintained, as previously described [58]. Briefly, eyes from 4-week-old TSP1^+/+^ and TSP1^−/−^ immorto mice were enucleated. Under a dissecting microscope in cold DMEM, the anterior eye was removed, followed by the lens, vitreous, retina, and the optic nerve, leaving only a tissue composed of RPE, choroid, and sclera. These tissues were pooled together, rinsed with DMEM, minced into small pieces in a tissue culture dish using sterilized razor blades, and digested in 5 mL of collagenase type I (1 mg/mL in serum free DMEM, Worthington, Lakewood, NJ, USA) for 45 min at 37 °C. Following digestion, DMEM with 10% FBS was added, and cells were pelleted. The cellular digests were then filtered through a double layer of sterile 40 µm nylon mesh (Sefar America Inc., Fisher Scientific, Hanover Park, IL, USA), centrifuged at 500× *g* for 10 min to pellet cells, washed twice with DMEM containing 10% FBS. Then, cells were suspended in a 1 mL medium (DMEM with 10% FBS) and incubated with magnetic beads pre-coated with anti-platelet endothelial cell adhesion molecule-1 (PECAM-1). After affinity binding, magnetic beads were washed six times with DMEM with 10% FBS, and the bound cells in the endothelial cell growth medium were plated into a single well of a 24-well plate pre-coated with 2 µg/mL of human fibronectin (BD Biosciences, Bedford, MA, USA). Endothelial cells were grown in DMEM media, supplemented with 10% FBS, 2 mM L-glutamine, 2 mM sodium pyruvate, 20 mM HEPES, 1% non-essential amino acids, 100 µg/mL streptomycin, 100 U/mL penicillin, freshly added heparin at 55 U/mL (Sigma, St. Louis, MO, USA), endothelial growth supplement 100 µg/mL (Sigma, St. Louis, MO, USA), and murine recombinant interferon-γ (R & D, Minneapolis, MN, USA) at 44 units/mL. Cells were maintained in 1% gelatin-coated 60 mm dishes at 33 °C with 5% CO_2_.

### 4.6. Fluorescein Isothiocyanate (FITC)-Dextran Permeability Assay

ARPE-19 cells were seeded on collagen/fibronectin coated membranes with 0.4 µm pores (Transwell; Corning Costar, Sigma-Aldrich, St. Louis, MO, USA). FITC-dextran flux permeability assays were performed, as previously described [9,11]. Briefly, cells were seeded until a complete confluent layer was formed, then cells were incubated in serum-free media for 24 h before Hcy treatment (20, 50, or 100 µM) (Sigma-Aldrich, St. Louis, MO, USA) of the upper chambers for 24 h, in the presence or absence of the NMDAR inhibitor MK801, followed by the addition of 10 µM FITC-dextran to the upper chambers. Aliquots were collected from the upper and lower chambers at 1, 3, or 6 h and then placed in a 96-well plate to measure the fluorescence intensity with a plate reader. The rate of diffusive flux (Po) FITC-dextran was calculated by the following formula [59]: Po = [(FA/∆t) VA]/(FLA). Where Po is in centimeters per second; FA is the lower chamber fluorescence; FL is the upper chamber fluorescence; ∆t is change in time; A is the surface area of the filter (in square centimeters); and VA is the volume of the lower chamber (in cubic centimeters).

### 4.7. Quantitative Reverse-Transcriptase Polymerase Chain Reaction (RT-q PCR) for NMDAR1

Total RNA was extracted from ARPE-19 cells and human neuroblastoma cells using TRIzolTM Reagent (Invitrogen, Eugene, OR, USA). Following RNA extraction and quantification, the iScript™ Synthesis kit (BioRad Laboratories, Hercules, CA, USA) was used for reverse transcription of 2 µg of the RNA. For the amplification of the produced cDNA, a gene-specific primer for NMDAR1, the absolute QPCR SYBR Green Fluorescein Mix (Thermo Scientific, Surrey, UK), and the BioRadiCycler (BioRad, Hercules, CA, USA) were used. The 18S gene was used as control for normalization. The primers used were NMDAR1 (NMDAR1 human F1: 5′ AAG CTG AGG GTG TGA AAC GG-3′, NMDAR1 human R1: 5′ GAG AGC CTG GAA ACT GGA CC-3′). Amplification parameters were as follows: 40 cycles of 95 °C for 30 s, 60 °C for 30 s, and 72 °C for 30 s. A melt curve analysis was performed to confirm the purity of the end products. Comparative CT method was used to obtain fold changes in the gene expression [11].

### 4.8. Western Blot Analysis

Western Blot analysis was used to detect NMDAR, HIF-1α, and VEGF in RPE cells and albumin in mice retina. After treatment of ARPE-19 with Hcy, the media was removed, and cells were lysed in RIPA buffer supplemented with 1:100 (*v*/*v*) of proteinase/phosphatase inhibitor cocktail (Thermo Scientific, Waltham, MA, USA). Likewise, primary RPE and mice retina were lysed in RIPA buffer supplemented with proteinase/phosphatase inhibitor cocktail. Then, cell lysates and tissue homogenate were centrifuged at 12,000× *g* at 4 °C for 30 min. Protein concentration was determined by BCA Protein Assay (Thermo Scientific, Waltham, MA, USA) and an equal amount of protein was boiled in a Laemmli sample buffer. Samples were subsequently subjected to gel electrophoresis on sodium dodecyl sulfate-polyacrylamide gel (SDS-PAGE) and the protein was blotted onto nitrocellulose membranes, which were further blocked using a 5% milk solution and then incubated with the following antibodies: NMDAR1 (Cell signaling, Danvers, MA, USA, Ca # 5704S), NMDAR2A (Cell signaling, Danvers, MA, USA, Ca # 4205s), NMDAR2B (Cell signaling, Danvers, MA. USA. Ca # 4207s), HIF-1α (Abcam, Cambridge, MA, USA, Cat # ab82832), VEGF (Thermofisher, Waltham, MA, USA, Cat #5-13182), Albumin (Bethyl, Montgomery, TX, USA), GAPDH (Sigma-Aldrich, St. Louis, MO, USA), and β-actin (Cell signaling, Danvers, MA. USA. Cat #937215). Then, blots were incubated with an appropriate peroxidase-conjugated secondary antibody and visualized with the enhanced chemiluminescence (ECL) western blot detection system (Thermo Scientific, Waltham, MA, USA). ImageJ software was used to determine the optical density of the bands.

### 4.9. Enzyme-Linked Immunosorbent Assay (ELISA)

An ELISA kit was used to further evaluate the effect of elevated Hcy on VEGF activation by measuring the level of VEGF in ARPE-19 treated with Hcy (20, 50, and 100 µM) for 24 h. After treatment, media was removed, followed by washing twice by PBS. Then, the cells were solubilized by using a cell extraction buffer and VEGF was measured in these cell lysates using a Human VEGF ELISA Kit (ab100662 ELISA Kit, Abcam, Cambridge, MA, USA). Absorbance was measured by a plate reader at 450 nm.

### 4.10. Immuno-Fluorescent Assessment

Primary Mouse Cells and Retina NMDAR1 and angiogenic factors (HIF-1α and VEGF) were assessed in primary RPE cells; Ki67 and ZO-1 were assessed in MCEC, and Isolectin-B4 in Retina. Cells were fixed in 4% paraformaldehyde for 10 min, washed with PBS, and blocked for one hour with Power Block (BioGenex, Fremont, CA, USA), Ca.#BS-1310-25. Then, cells were incubated with anti-NMDAR1 (Cell signaling, Danvers, MA, USA, Ca#5704S), anti-HIF-1α (Abcam, Cambridge, MA, USA, Cat#ab82832), anti-VEGF (Thermofisher, Waltham, MA, USA, Cat#5-13182), and anti-Ki67 (Abcam, Cambridge, MA, USA, Cat#ab15580) for 3 h at 37 °C. After that, cells were washed 3 times with PBS containing 0.3% Triton-X. After the last wash, cells were incubated with the appropriate secondary antibodies (Alexafluor and Texas red avidin, Invitrogen, Eugene, OR, USA), and cover-slipped with Fluoroshield containing DAPI (Sigma-Aldrich Chemical Corp., St. Louis, MO, USA) to label the nuclei. An Axioplan-2 fluorescent microscope (Carl Zeiss, Göttingen, Germany) equipped with a high-resolution microscope (HRM) camera was used to capture images using the Zeiss Axiovision digital image processing software (version 4.8). Additionally, NMDAR expression was assessed in retinal RPE flat-mounts, isolated from mice injected intravitreally with Hcy (WT and *NMDAR*^−/−*R*^ mice). RPE flat-mounts were performed following our published method [53].

NMDAR expression and isolectin-B4 (marker for blood vessel) were assessed using immunofluorescence in RPE flat-mounts and retinal frozen sections (sections were prepared from wild-type and Hcy-injected mice), as mentioned in our published method [11]. Briefly, flat-mounts and frozen sections were fixed with 4% paraformaldehyde and blocked with Power Block, then incubated with primary antibody for 3 h at 37 °C. These were subsequently washed and incubated with secondary antibody (Alexafluor and Texas red avidin, Invitrogen, Eugene, OR, USA). Next, mounts and sections were cover-slipped with Fluoroshield containing DAPI (Sigma-Aldrich, St. Louis, MO, USA) to label the nuclei. An Axioplan-2 fluorescent microscope (Carl Zeiss, Göttingen, Germany) equipped with a high-resolution microscope (HRM) camera was used to capture images using the Zeiss Axiovision digital image processing software (version 4.8). Samples were representative of at least three mice from each IF experiment.

### 4.11. Optical Coherence Tomography (OCT) and Fluorescein Angiography (FA)

To evaluate fluorescein leakage and retinal structure after the intravitreal injection of Hcy into wild-type and *NMDAR*^−/−*R*^ mice, and to evaluate fluorescein leakage and retinal structure after 2 days from laser induction of CNV in Hcy intravitreally injected mice (wild-type and *NMDAR*^−/−*R*^), OCT and FA were performed according to our published methods, with some modifications [9,18,23,24]. Briefly, mice were injected intravitreally with Hcy (200 µM), and OCT and FA were performed after 48 h. For the laser experiment, laser induction was performed 5 days after Hcy injection; 2 days after laser induction, OCT and FA were performed. The intravitreal injection was performed following our previously published protocol [9,18,23,24]. Briefly, a dose of 1 μL l-homocysteine thiolactone hydrochloride (Sigma-Aldrich, St. Louis, MO, USA) was utilized for intravitreal injection to avoid an uncontrolled increase of the intraocular pressure (assuming a vitreous volume of mouse eye is ~10 μL). l-homocysteine thiolactone hydrochloride was liquified in water and a working solution of 10 × was obtained by diluting a dose of 1 μL from the stock solution (200 mM) in 100 μL of the PBS, followed by the intravitreal injection of 1 μL of this working solution, aiming for a vitreal concentration of 200 μM of Hcy thiolactone. The mice were anesthetized using 2% isoflurane, and the eye pupils were dilated by using a 1% tropicamide eye drop. Then, each mouse was placed on the imaging platform of the Phoenix Micron III retinal imaging microscope supplemented with an OCT imaging device (Phoenix Research Laboratories, Pleasanton, CA, USA). Lubricant gel was applied to keep the eye moist during imaging. For FA, mice were injected with (10–20 µL, IP) 10% fluorescein sodium (Apollo Ophthalmics, Newport Beach, CA, USA), followed by the rapid acquisition of fluorescent images for ~5 min. Fluorescein leakage manifests as indistinct vascular borders progressing to diffusely hazy fluorescence.

### 4.12. Laser Induction Using Phoenix MICRON Image-Guided Laser System

The mice were placed on the imaging platform Phoenix Micron IV retinal imaging microscope after being anesthetized and prepared, as mentioned in the FA and OCT imaging. The fundus was viewed with the micron IV fundus camera, and laser photocoagulation was induced using the image-guided laser system (Micron IV, Phoenix Research Laboratories, Pleasanton, CA, USA). The fundus image as well as the aiming beam could be observed on the monitor screen. Four–five laser burns at an equal distance from the optic nerve were induced one by one in each eye by a green Argon laser pulse, with a wavelength of 532 nm, a duration of 70 ms, and power levels from 250 mW to 260 mW. Successful laser burns were confirmed by the appearance of white bleep with grey outline, indicating the break of Bruch’s Membrane. After laser photocoagulation, the mice were then placed under an infra-red warming lamp until they awakened.

### 4.13. Measuring Retinal Thickness

Spectral domain OCT with the guidance of a bright-field live fundus image was performed with the image-guided OCT system (Phoenix Research Labs, Pleasanton, CA, USA) according to the manufacturer’s instructions and using the StreamPix 6 software version 7.2.4.2 (Phoenix Research Labs, Pleasanton, CA, USA) to generate fundus images and OCT scans. Using the InSight software, version 2.1.7237, (Phoenix Research Labs, Pleasanton, CA, USA), the borderlines between the retinal layers were defined on the OCT pictures. These borderlines were initially indicated automatically by the software; they were then manually corrected by the researchers, when necessary. Next, the distance (in µm) between each borderline was calculated using the InSight software at 300 consecutive points throughout the borderline, and the average of these data was defined as the thickness of the respective layer (NFL + IPL, INL, OPL, ONL, outer+ inner segments, RPE, and Choroid).

### 4.14. Data Analysis

Results were conveyed as mean ± SD. Assessment of differences among experimental groups was performed using the two-tailed *t* test or one-way analysis of variance (ANOVA). When statistical differences were detected using ANOVA, Tukey’s post hoc test was performed to determine which groups differed. Statistical significance was considered at a confidence level of *p* < 0.05. HIF1 alpha and Ki67 color staining intensity are available in the supplementary data (Figure S1).

## Data Availability

Western blot data was available as supplementary material. The rest of the data is available on reasonable request from the corresponding author.

## Supplementary Materials

The following are available online at www.mdpi.com/article/10.3390/ijms22179356/s1.
