# Supplementary figures and images for: Implication of N-Methyl-d-Aspartate Receptor in Homocysteine-Induced Age-Related Macular Degeneration
Source: Int J Mol Sci. 2021 Aug 28;22(17):9356. doi: 10.3390/ijms22179356 (PMC8431693; doi:10.3390/ijms22179356)

## Slide 1
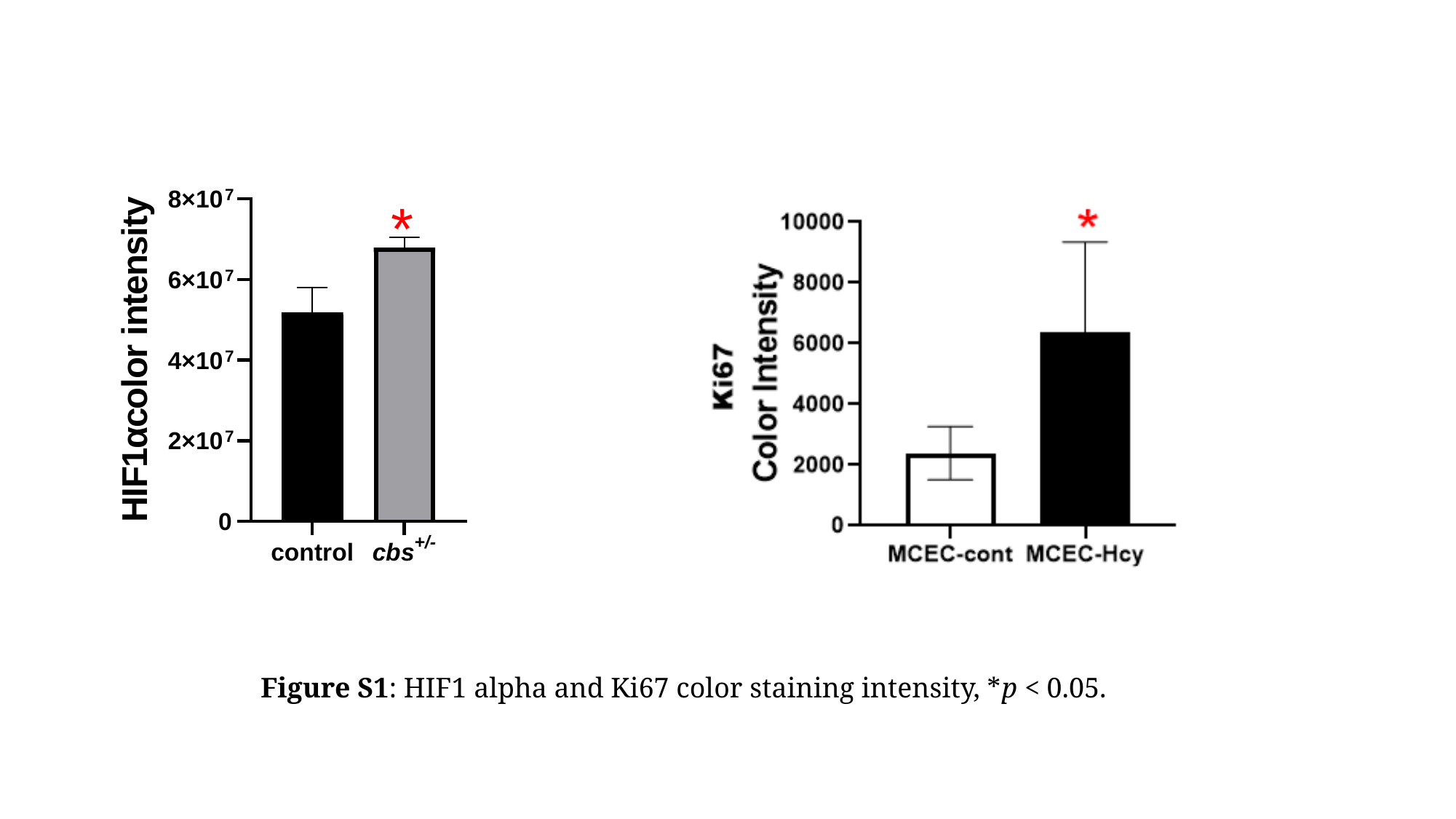

Figure S1: HIF1 alpha and Ki67 color staining intensity, *p < 0.05.

Supplement: Supplementary file 1 [file ijms-22-09356-s001.zip › ijms-1340927-supplementary.pptx]
